# GAS-Luc2 Reporter Cell Lines for Immune Checkpoint Drug Screening in Solid Tumors

**DOI:** 10.3390/cancers16111965

**Published:** 2024-05-22

**Authors:** Hyeyoun Chang, John G. Foulke, Luping Chen, Fang Tian, Zhizhan Gu

**Affiliations:** American Type Culture Collection (ATCC), Manassas, VA 20110, USA

**Keywords:** IFNγ, GAS-Luc2, immune checkpoint, solid tumor, drug screening, PD-L1, CD155, B7-H3/CD276, artificial antigen-presenting cell (aAPC)

## Abstract

**Simple Summary:**

Despite the recent studies emphasizing the importance of the interferon gamma receptor (IFNγR) pathway in T cell-mediated cytotoxicity against solid tumors, full human ex vivo immune checkpoint drug screening remains a challenge. We employed an engineered gamma interferon activation site response element luciferase reporter (GAS-Luc2) for immune checkpoint drug screening in diverse ex vivo T cell–solid tumor cell co-culture systems. Three GAS-Luc2 reporter tumor cell lines endogenously expressing various immune checkpoints were engineered to produce quantifiable bioluminescence signal in the presence of an immune checkpoint inhibitor where activated T cells release IFNγ. These reporter cell lines also detected paracrine IFNγ signaling for immune checkpoint-targeted ADCC drug screening. Advancement into an artificial antigen-presenting cell line (aAPC) significantly enhanced T cell signaling for improved performance in these ex vivo immune checkpoint drug screening platforms.

**Abstract:**

Recent studies highlight the integral role of the interferon gamma receptor (IFNγR) pathway in T cell–mediated cytotoxicity against solid but not liquid tumors. IFNγ not only directly facilitates tumor cell death by T cells but also indirectly promotes cytotoxicity via myeloid phagocytosis in the tumor microenvironment. Meanwhile, full human ex vivo immune checkpoint drug screening remains challenging. We hypothesized that an engineered gamma interferon activation site response element luciferase reporter (GAS-Luc2) can be utilized for immune checkpoint drug screening in diverse ex vivo T cell–solid tumor cell co-culture systems. We comprehensively profiled cell surface proteins in ATCC’s extensive collection of human tumor and immune cell lines, identifying those with endogenously high expression of established and novel immune checkpoint molecules and binding ligands. We then engineered three GAS-Luc2 reporter tumor cell lines expressing immune checkpoints PD-L1, CD155, or B7-H3/CD276. Luciferase expression was suppressed upon relevant immune checkpoint–ligand engagement. In the presence of an immune checkpoint inhibitor, T cells released IFNγ, activating the JAK-STAT pathway in GAS-Luc2 cells, and generating a quantifiable bioluminescent signal for inhibitor evaluation. These reporter lines also detected paracrine IFNγ signaling for immune checkpoint-targeted ADCC drug screening. Further development into an artificial antigen-presenting cell line (aAPC) significantly enhanced T cell signaling for superior performance in these ex vivo immune checkpoint drug screening platforms.

## 1. Introduction

Cancer immunotherapies targeting immune checkpoints, such as immune checkpoint inhibitors (ICIs), antibody-dependent cellular cytotoxicity (ADCC), and antibody–drug conjugates (ADCs), have shown tremendous success in the treatment of solid tumors such as skin, lung, breast, renal, and liver cancers [1]. However, the built-in complexity of immunological models, variable drug responses among different cancer types, and substantial financial burdens have challenged the development and application of these novel immunotherapies [2]. While immuno-oncology studies have made significant progress by using syngeneic mouse models or humanized mouse models, the inevitable differences between the mouse immune system, or a hybrid human/mouse immune system, and the human immune system remain a concern [3]. Hence, interest regarding ex vivo and in vitro fully human immuno-oncology assays has rapidly increased in recent years; many of which focus on co-culture assays utilizing various primary human immune cells that take part in killing human cancer cells in vivo [4,5]. Nonetheless, the prevailing assays in this category largely employ an artificial system with engineered ectopic overexpression of immune checkpoint molecules and a T cell activation signaling reporter gene in immortalized T cells, such as an engineered Jurkat T cell line (CD4+, non-cytotoxic) with stable expression of human PD-1 and a nuclear factor of activated T cells (NFAT)-driven luciferase reporter [6]. More physiologically relevant immuno-oncology reporter assays are urgently needed for better modeling of the in vivo immune response to cancer, such as CD8+ cytotoxic T cell-mediated cancer cell killing events, and for immuno-oncology drug discovery and development.

Cancer immunotherapy has been an effective therapeutic approach in many hematopoietic malignancies, especially for the PD1/PD-L1 and CTLA-4 blockade therapies [7,8]. Additionally, immune checkpoint blockade therapies for TIGIT/CD155 as well as targeting B7-H3 have also been investigated and have shown promise in use as immunotherapies [9,10,11,12]. However, the success rate of these therapies is significantly lower in solid tumors [13,14]. Recent studies have demonstrated, both in vitro and in vivo, that the interferon-γ receptor (IFNγR) JAK-STAT signaling pathway is required for T cells to kill cancer cells in solid tumors but not in liquid cancers [15]. Further studies suggested that IFNγ plays a central role not only in direct T cell killing of tumor cells but also in indirect T cell-mediated cytotoxicity via myeloid phagocytosis in the solid tumor microenvironment [16]. Meanwhile, the cell signaling reporter system containing a GAS-response element upstream of the luciferase gene (e.g., Firefly luciferase/Luc2) has been widely used for detecting inter-cell IFNγ signaling [17,18,19]. Hence, we utilized the demonstrated IFNγ-IFNγR JAK-STAT GAS-Luc2 reporter system for engineering solid tumor cell lines that are capable of robustly reporting the activity of IFNγ from any activated IFNγ-producing immune cells. Because the reporter system was integrated into tumor cells instead of a T cell line [6], we were able to use primary immune cells that included—but were not limited to—CD8+ cytotoxic T cells, CD4+ helper T cells (Th1), and CD56+ NK cells. This allowed the system more versatility in terms of primary immune cell selection during their interaction with solid tumor cells.

To precisely select cell lines naturally expressing specific immune checkpoint molecules for building solid tumor–cytotoxic T cell inter-cell signaling reporter assay systems to facilitate cancer immunotherapy drug screening, we initiated the study by performing protein profiling of immune checkpoint molecules and their paired ligands. We performed flow cytometry-based immune checkpoint protein profiling in over 50 tumor cell lines and over 10 immune cell lines available at ATCC^®^ (Manassas, VA, USA). Based on the proteomics data, the three cell lines: HCC827, MG-63, and NCIH1650 were selected for their high endogenous expression of the immune checkpoint proteins: PD-L1, CD155, and B7-H3, respectively. These immune checkpoint reporter systems were designed so that the naturally high expression of the immune checkpoint molecules on the surface of the reporter cells would facilitate the immune checkpoint inhibitor drug candidate binding, promoting immune cell activation and subsequent release of IFNγ by the immune cells; thereby resulting in intracellular IFNγ-IFNγR JAK-STAT GAS signaling activation and luciferase expression by the reporter cells (Graphical Abstract). Lastly, we engineered an additional GAS-Luc2-aAPC reporter cell line expressing anti-CD3/CD28 to enhance the recognition of tumor cells by ex vivo primary immune cells.

## 2. Materials and Methods

### 2.1. Cell Culture

Cell lines were cultured according to the handling procedures described on the respective ATCC^®^ (Manassas, VA, USA) product sheets. The following cell lines were cultured: bladder cancer cell lines (5637, HT-1197, HT-1376, RT4, TCCSUP), brain cancer cell lines (SK-N-BE(2), U-87 MG, U-87 MG-Luc2), breast cancer cell lines (AU565, BT-20, DU4475, HCC38, MCF7, MCF7-Luc2, MDA-MB-231, MDA-MB-468, T-47D), bone cancer cell lines (HOS, MG-63, Saos-2, U-2 OS)), colon cancer cell lines (Caco-2, HCT-15, LoVo), head and neck cancer cell lines (A-253, FaDu, FaDu-Luc2), liver cancer cell lines (CA3 [HPEG2/C3A], SK-HEP-1), lung cancer cell lines (A549, Calu-1, NCI-H650 [H-1650, H1650], NCI-H229 [H226], NCI-H441 [H441], NCI-H460 [H460], HCC827, NCI-H1299, NCI-H1975 [H1975], NCI-H596 [H596]), melanoma cell lines (A-375 [A375], A375-KRAS, A375-KRAS-Luc2, RPMI-7951, SH-4, SK-MEL-24), an ovarian cancer cell line (ES-2), pancreatic cancer cell lines (ASPC-1. PANC-1, PANC 10.05), prostate cancer cell lines (PC-3, PC-3-Luc2), other skin cancer cell lines (A-431, A-431-Luc2), a uterine cancer cell line (HEC-1-A), T cell lines (Jurkat E6-1, TALL-104, MOLT-2. HH, HuT 78, SUP-T1, HM2, MJ [g11], CCRF-CEM), primary human CD4+ and CD8+ T cells, and CD56+ NK cells. After viral transduction, GAS-Luc2 cell lines were selected and maintained in the same culture media as the parental cell lines but with additional 0.25–1 µg/mL puromycin dihydrochloride (Sigma-Aldrich^®^, St. Louis, MO, USA). The HCC827-GAS-Luc2-aAPC cell line was maintained in the same culture media as the HCC827-GAS-Luc2 cell line.

### 2.2. Cell Surface Protein Profiling via Flow Cytometry

For the flow cytometry analyses, cells were stained with fluorescently labeled antibodies specific for inhibitory or co-stimulatory immune checkpoint molecules that have been well characterized in the literature (Supplemental Figure S1). Cell staining was performed in Fetal Bovine Serum Stain Buffer (BD Biosciences™, Franklin Lakes, NJ, USA) with the antibodies diluted to the manufacturers’ recommended concentrations for 30 min on ice in the dark. Cells were then fixed by the addition of an equal volume of Fixation Buffer (BD Biosciences™, Franklin Lakes, NJ, USA). The cancer cells were tested both in the presence and absence of overnight IFNγ stimulation (100 ng/mL). For each sample, 10,000 stained cells were captured by the Accuri C6 Plus flow cytometer (BD Biosciences™, Franklin Lakes, NJ, USA) and the results were analyzed by the FlowJo^®^ software version v10.9.0. The measurements were used to generate the heat maps via Microsoft Excel Version 2308.

### 2.3. Plasmid Transduction and Single Cell Cloning

For establishing GAS-Luc2 cell lines, candidate cell lines were seeded at 1 × 10^5^ cells/well into a 12-well culture plate and transduced with lentiviral-GAS-Luc2 plasmids in the presence of 50 μg/mL protamine sulfate (Sigma^®^) for 24 h. Transduction was stopped by replacing the medium with fresh culture medium. The lentiviral-GAS-Luc2 plasmids were generated by replacing the EF-1 alpha promoter with a GAS response element in a lentiviral-luciferase plasmid. The transduced cells were enriched by puromycin selection and evaluated by IFNγ stimulation as described in the next paragraph. Following the luciferase expression assessment, single cells were isolated by automated cell sorting (Sony SH800, Sony Biotechnology, San Jose, CA, USA) into 96-well plates and expanded for approximately 10–14 days until their confluency reached 70%. The growing single-cell clones were then subcultured and stimulated with IFNγ again. The clone that yielded the highest luminescence signal upon IFNγ stimulation was chosen for the subsequent T cell-conditioned media stimulation and immune cell co-culture experiments.

### 2.4. Interferon Gamma Stimulation

To assess the luminescence signal intensity upon stimulation, IFNγ (R&D Systems^®^, Minneapolis, MN, USA) was added to each GAS-Luc2 engineered cell line. The cells were seeded in 100 µL culture media on a 96-well plate (Corning^®^, Tewksbury, MA, USA) at the seeding density of 5.0 × 10^3^ cells/well. Final concentrations of 0, 0.01, 0.1, 1, 10, 100, and 1000 ng/mL IFNγ were added to the cells, followed by overnight incubation at 37 °C in 5% CO_2_; experimental controls included media only and untreated cells. The next day, an equal volume of Bright-Glo™ Luciferase Assay Reagent (Promega^®^, Madison, WI, USA) was added to the cells and the plate was incubated on a shaker for 8 min. The assay mixture was transferred to an opaque 96-well plate (Corning^®^, Tewksbury, MA, USA)) for luminescence detection. The luminescence signals were measured by a SpectraMax^®^ i3x (Molecular Devices^®^, San Jose, CA, USA). Cells were evaluated based on the average fold increase of relative luminescence (RLU) of IFNγ-stimulated cells relative to average RLUs from untreated controls. Statistical calculation was carried out using multiple *t*-test.

### 2.5. T Cell-Conditioned Media Stimulation

Primary human CD8+ cytotoxic T cells (ATCC^®^, Manassas, VA, USA) were cultured in RPMI-1640 (ATCC^®^, Manassas, VA, USA) with 10% heat-inactivated FBS (Gibco^®^, Waltham, MA, USA) and 50 U/mL interleukin-2 (Miltenyi^®^ Biotec, Gaithersburg, MD, USA). On day 0, half of the CD8+ cytotoxic T cells were activated using a T Cell Activation/Expansion Kit (Miltenyi^®^ Biotec, Gaithersburg, MD, USA) following the manufacturer’s instructions. The remaining cells were centrifuged at 400× *g* for 5 min to remove the old medium, and were then resuspended in fresh culture medium at a seeding density of 2.0 × 10^6^ cells/mL. On day 3, the activated and non-activated T cells were harvested by centrifugation, and the supernatants were collected as activated and non-activated CD8+ cytotoxic T cell-conditioned media, respectively. Upon preparation of the T cell-conditioned media, each GAS-Luc2-engineered monoclonal cell line was harvested and seeded in 100 µL culture media on a 96-well plate at the seeding density of 2.0 × 10^4^ cells/well. The cells were incubated at 37 °C in 5% CO_2_ for 1 h to attach to the plates. After the cells settled down, an equal volume of the T cell-conditioned media was added to the cells. The cells were then incubated overnight before proceeding to the luciferase assay as described above.

### 2.6. Cancer Cell and Immune Cell Co-Culture

The monoclonal GAS-Luc2 cell lines were co-cultured with primary human immune cells, including CD8+ cytotoxic T cells, CD4+ helper T cells, and CD56+ NK cells. The co-culture methods were optimized for each type of antibody used. For the co-culture with the anti-PD-L1 antibody atezolizumab (Invivogen^®^, San Diego, CA, USA, hpdl1-mab1, MOA: PD-L1 blockade), the HCC827-GAS-Luc2 cells were added to a 96-well plate at the seeding density of 1.0 × 10^4^ cells/well and pre-incubated for 15 min with five different concentrations (0, 1, 10, 100, or 1000 ng/mL) of atezolizumab or an isotype control (Invivogen^®^, San Diego, CA, USA, bgal-mab1) before primary human CD8+ cytotoxic T cells were added to the GAS-Luc2 cells at a 1:1, 1:2, 1:5, or 1:10 ratio of target cells to effector cells. The cells were co-cultured at 37 °C in 5% CO_2_ for 24 h. Additionally, 2-, 4-, and 6-h co-cultures were performed with a 1:10 ratio of HCC827-GAS-Luc2 cells to CD8+ T cells. Utilizing the same method, HCC827-GAS-Luc2 cells were also co-cultured with CD4+ helper T cells for 24 or 48 h with a 1:1 ratio of target to effector cells. The co-culture experiments were followed by the luciferase assay, as described above. For the co-culture with the anti-TIGIT antibody tiragolumab (ProSci^®^, Poway, CA, USA, 10-856, MOA: TIGIT blockade), CD4+ helper T cells were first added to a 96-well plate at a seeding density of 1.0 × 10^4^ cells/well and pre-incubated for 15 min with five different concentrations (0, 1, 10, 100, or 1000 ng/mL) of tiragolumab or an isotype control (BioLegend^®^, San Diego, CA, USA, 403501) before MG-63-GAS-Luc2 cells were added to the CD4+ helper T cells at a 1:1 ratio of target to effector cells. The cells were co-cultured for 24 or 48 h and subsequently evaluated via the luciferase assay. For the co-culture with the anti-B7-H3 antibody enoblituzumab (ProSci^®^, Poway, CA, USA, 10-755 MOA: B7-H3 engaged ADCC), H1650-GAS-Luc2 cells were seeded to a 96-well plate at a seeding density of 1.0 × 10^4^ cells/well and pre-incubated for 15 min with five different concentrations (0, 1, 10, 100, or 1000 ng/mL) of enoblituzumab or an isotype control (Biolegend^®^, San Diego, CA, USA, 403501) before primary human CD56+ NK cells were added to the GAS-Luc2 cells at a 2:1 ratio of target to effector cells. The cells were co-cultured for 24 or 48 h prior to the evaluation by the luciferase assay.

### 2.7. CD8+ Cytotoxic T Cell Co-Culture on Cancer Cell Viability

After 24 h of co-culture of HCC827-GAS-Luc2 cells and CD8+ cytotoxic T cells with a 1:5 ratio of target to effector cells in the presence of the anti-PD-L1 antibody or the isotype control as described above, the culture medium was carefully removed by pipetting and the cells were gently washed three times with PBS to remove any floating cells and cell debris. The cells were then stained with 0.2% trypan blue (Gibco^®^, Waltham, MA, USA) and six brightfield images were taken for each condition under an Eclipse TE300 inverted microscope (Nikon^®^, Melville, NY, USA) within 3 to 5 min of mixing with trypan blue. The number of unstained live cells in these images was manually counted. The percent viability for each condition was calculated as the average number of live cells for the condition divided by the average number of live cells observed in no-antibody control multiplied by 100%.

### 2.8. CD4+ Helper T Cell Subset Phenotyping

Primary human CD4+ helper T cells (ATCC^®^, Manassas, VA, USA) were washed and re-suspended in cold Fetal Bovine Serum Stain Buffer (BD Biosciences™, Franklin Lakes, NJ, USA) before they were placed at the density of 1.0 × 10^6^ cells/well in a V-bottom 96-well plate (Thermo Fisher Scientific^®^, Waltham, MA, USA). The experimental sample cells were stained with anti-CD3-VioBlue (clone BW264/56), anti-CD4-FITC (clone RPA-T4), anti-CXCR3-PE-Vio (clone REA232), anti-CCR4-APC (clone REA279), and anti-CCR6-APC-Vio770 (clone REA190) antibodies on ice for 30 min in the dark at the concentrations recommended by the manufacturers (Supplemental Figure S1). The control samples included unstained cells, a fluorescence-minus-one control for each fluorescently conjugated antibody, and UltraComp eBeads Plus (Invitrogen^®^, Waltham, MA, USA) for single-color compensation controls stained according to the manufacturer’s recommendation. After staining, the cells were washed twice with Stain Buffer, then fixed by the addition of an equal volume of Fixation Buffer (BD Biosciences™, Franklin Lakes, NJ, USA) and Stain Buffer. The data were collected by CytoFLEX (Beckman Coulter^®^, Brea, CA, USA), andthe data analysis—including compensation matrix generation and sequential gating—was performed by FlowJo^®^ software version v10.9.0.

### 2.9. Generation of Artificial Antigen-Presenting Cell Line

To generate an aAPC that stably expresses anti-CD3 and anti-CD28, HCC827-GAS-Luc2 cells were lentivirally transduced with two CSRs (chimeric stimulatory receptors), an anti-CD3 single-chain variable fragment (scFv) with a GFP reporter, and an anti-CD28 scFv with a mCherry reporter. The OKT3 clone-derived anti-CD3 scFv and the 9.3 clone-derived anti-CD28 scFv constructs were linked with a CD8α hinge and transmembrane domain followed by glycine/serine linker and GFP or mCherry, respectively [20,21]. The HCC827-GAS-Luc2 cells were seeded at 1 × 10^5^ cells/well into a 12-well culture plate and the lentiviral plasmids (VectorBuilder™, Chicago, IL, USA) were added to the culture medium and incubated with 50 μg/mL protamine sulfate (Sigma^®^) for 24 h at 37 °C in 5% CO_2_. Transduction was stopped by replacing the medium with fresh culture medium. The newly generated aAPCs were stabilized for two weeks then bulk sorted (Sony^®^ SH800, Sony Biotechnology, San Jose, CA, USA) based on their positive GFP and mCherry expression, which was separately confirmed by flow cytometry (Beckman Coulter^®^, Brea, CA, USA, CytoFLEX) at one-week post-sorting (Supplemental Figure S7).

### 2.10. Quantification and Statistical Analysis

All experiments have been repeated at least three times. The statistical significance of the differences between the groups was analyzed using two-tailed paired *t*-test, multiple *t*-test, or one-way ANOVA. A *p*-value <0.05 was considered statistically significant. Statistical analyses were performed with GraphPad Prism (La Jolla, CA, USA) software version v9.0.0.

## 3. Results

### 3.1. Protein Profiling of Cancer Cell Lines for Immune Checkpoint Molecule Ligand Expression

**Figure 1:**
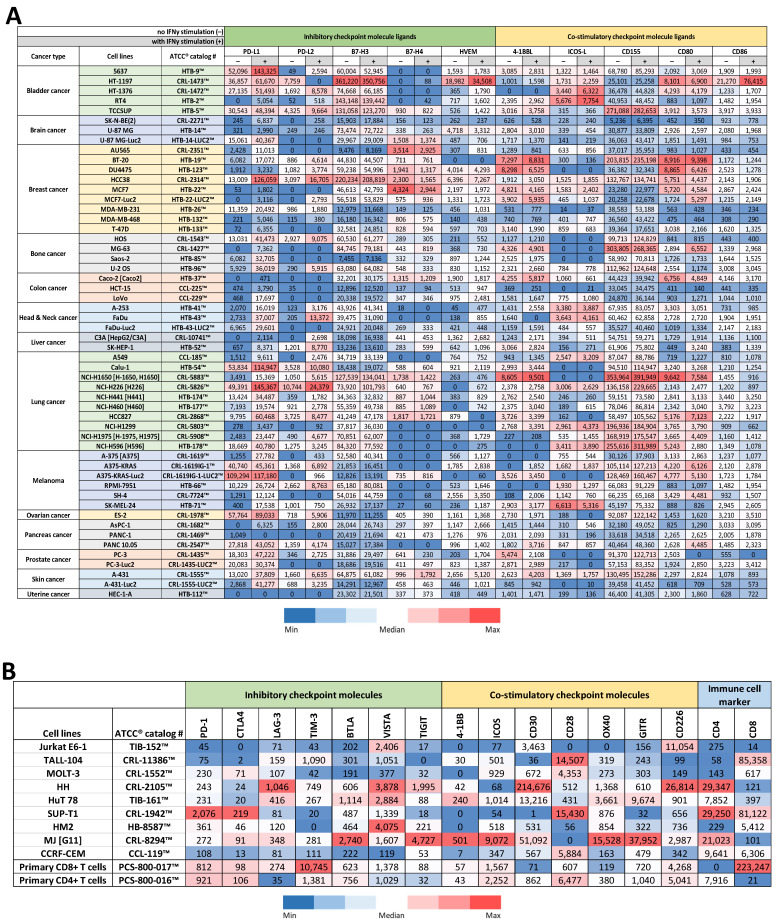
Protein profiling of immune checkpoint molecule expression in selected human cancer cell lines, T cell lines, and primary T cells. (**A**) A heat map was generated to compare the expression levels of both inhibitory and co-stimulatory immune checkpoint molecule ligands in selected cancer cell lines. Protein expression levels in cancer cell lines under basal (−) and 100 ng/mL IFNγ stimulated (+) conditions were profiled via flow cytometry. (**B**) An additional heat map was generated to compare the expression levels of inhibitory immune checkpoint molecules, co-stimulatory immune checkpoint molecules, and T cell markers in the selected cell lines. Table values represent median fluorescence intensity (MFI) sample values subtracted by isotype control MFI. Values of each Excel sheet column were color coded separately to avoid cross comparison between columns as different antibodies targeting their own specific antigen were used in each column of the flow cytometry protein profiling experiments.

Over 50 human cancer cell lines of various origins available at ATCC^®^ were collected for protein profiling via flow cytometry for inhibitory and co-stimulatory immune checkpoint molecule ligands. The checkpoint ligands tested were PD-L1, PD-L2, B7-H3, B7-H4, and HVEM for inhibitory ligands; and 4-1BBL, ICOS-L, CD155, CD80, and CD86 for co-stimulatory ligands. The protein profiling was performed with or without the stimulation of IFNγ (Figure 1A). While the expression levels of checkpoint ligands mostly differed from one cell line to another, some cell lines of the same origin shared similar expression patterns. For instance, four out of five bladder cancer cell lines showed minimal expression of B7-H4; all brain cancer cell lines and colon cancer cell lines tested expressed a low level of PD-L2; and bone cancer cell lines and lung cancer cell lines demonstrated elevated levels of CD155 as compared to the cell lines from other origins. The pancreatic cancer cell lines tested showed high levels of CD86 expression, but low levels of B7-H3 and CD155 expression. There were also variations between cell lines, where some cell lines expressed overall high levels of checkpoint ligands while other cell lines continuously expressed low levels of checkpoint ligands. For example, the SK-N-BE (2) brain cancer cell line expressed very low levels of all tested checkpoint ligands, while the HCC38 breast cancer cell line expressed high levels of almost all tested checkpoint ligands. Additionally, IFNγ stimulation did not necessarily increase or decrease the expression level of these immune checkpoint molecule ligands. In some cell lines, IFNγ stimulation significantly increased the expression of certain checkpoint ligands while in others the change was minimal, or the expression decreased. Representative flow cytometry raw data of high immune checkpoint molecule ligand expression are shown in Supplemental Figure S2.

### 3.2. Protein Profiling of T Cell Lines & Primary T Cells for Immune Checkpoint Molecule Expression

Similar protein profiling by using the flow cytometry method was conducted with nine human T cell lines and primary human CD8+ and CD4+ T cells available at ATCC^®^. Cells were profiled for both inhibitory checkpoint molecules, such as PD-1, CTLA4, LAG-3, TIM-3, BTLA, VISTA, and TIGIT, and co-stimulatory checkpoint molecules, such as 4-1BB, ICOS, CD30, CD28, OX40, GITR, and CD226. Evaluation of CD8 and CD4 expression levels were included (Figure 1B). While primary human CD8+ and CD4+ T cells expressed moderately high levels of checkpoint molecules, most of these expressions noticeably declined in many of the T cell lines tested. However, seven out of nine tested cell lines showed a significant increase in at least one of the checkpoint molecules tested. The Jurkat E6-1 T cell line expressed higher levels of VISTA and CD226; the TALL-104 cell line expressed higher level of CD28; the HH cell line expressed higher levels of LAG-3, VISTA, TIGIT, CD30, and CD226; the HuT 78 cell line expressed higher levels of LAG-3, BTLA, VISTA, 4-1BB, CD30, OX40, and GITR; the SUP-T1 cell line expressed higher levels of PD-1, CTLA4, and CD28; the HM2 cell line expressed higher level of VISTA; and the MJ [G11] cell line expressed higher levels of BTLA, TIGIT, 4-1BB, ICOS, CD30, OX40, and GITR as compared to the expression levels of the primary cells. It is also worth noting that four out of the seven cell lines showed higher CD4 molecule expression than primary human CD4+ T cells, but all the T cell lines demonstrated significantly lower CD8 molecule expression than primary human CD8+ T cells. Primary human CD8+ T cells also showed exceptionally high level of TIM-3, which was downregulated in all the T cell lines tested. Representative flow cytometry raw data of high immune checkpoint molecule expression are shown in Supplemental Figure S3.

### 3.3. Selection of Candidate Reporter Cell Lines

Based on the protein profiling results, cancer cell lines that were found to endogenously express high levels of certain immune checkpoint markers were selected as cancer reporter cell line candidates. The selected cell lines were virally transduced with a Lenti-Luc2P plasmid containing a gamma interferon activation site (GAS)-response element to monitor the activity of IFNγ-induced signal transduction pathways via luciferase expression. Following viral transduction and antibiotic selection, the GAS-Luc2-engineered cell line candidates at the stage of multi-clone pool were assessed using an IFNγ cytokine stimulation assay (Supplemental Figure S4). GAS-Luc2-engineered cell lines with high expression of PD-L1 (CALU-1, TCCSUP, and HCC827), CD155 (MG-63 and NCI-H596), or B7-H3 (NCI-H1650 and TCCSUP) were evaluated under various concentrations of IFNγ stimulation. After a 24-h incubation period, cells were assessed for luminescence signal intensity. All cell lines expressed increased luminescence with increased administration of IFNγ, but the cell lines HCC827-GAS-Luc2, MG-63-GAS-Luc2, and NCI-H1650-GAS-Luc2 exhibited the highest luminescence values within their respective groups, showing a 150-, 150-, and 50-fold increase in RLU values relative to untreated controls under 1000 ng/mL IFNγ administration, respectively. In addition, the HCC827-GAS-Luc2 candidate cell line demonstrated the highest sensitivity to IFNγ stimulation, exhibiting increased luminescence signal with the concentration as low as 0.01 ng/mL. Because of their higher sensitivity and higher signal intensity, the HCC827-GAS-Luc2, MG-63-GAS-Luc2, and NCI-H1650-GAS-Luc2 cell lines were selected for further study as a PD-L1, CD155, and B7-H3 checkpoint inhibitor cancer cell reporters, respectively.

### 3.4. Comparisons of Physical Properties of Reporter Cell Lines to Parental Cell Lines

Upon completing single-cell sorting and single-cell clonal expansion, additional studies were conducted to ensure that the physical properties of the monoclonal GAS-Luc2-engineered cell lines were comparable to their parental counterparts. Flow cytometry analysis was conducted to compare the respective checkpoint molecule expression between GAS-Luc2-engineered cells and parental cells to assess if the engineered cells retained the high expression of PD-L1, CD155, or B7-H3 (Supplemental Figure S5). All three engineered cell lines were shown to maintain a similar level of expression for the respective checkpoint molecule as their parental counterparts. Moreover, microscopy analysis revealed that the morphology of the parental cell lines was maintained in all three engineered cell lines; no discernable physical differences were identified (Supplemental Figure S6). Growth rate analysis of the three engineered cell lines also showed that the change in their growth rate was within ±20% of that of their parental.

### 3.5. IFNγ and T Cell-Conditioned Media Stimulation of Reporter Cell Lines

**Figure 2:**
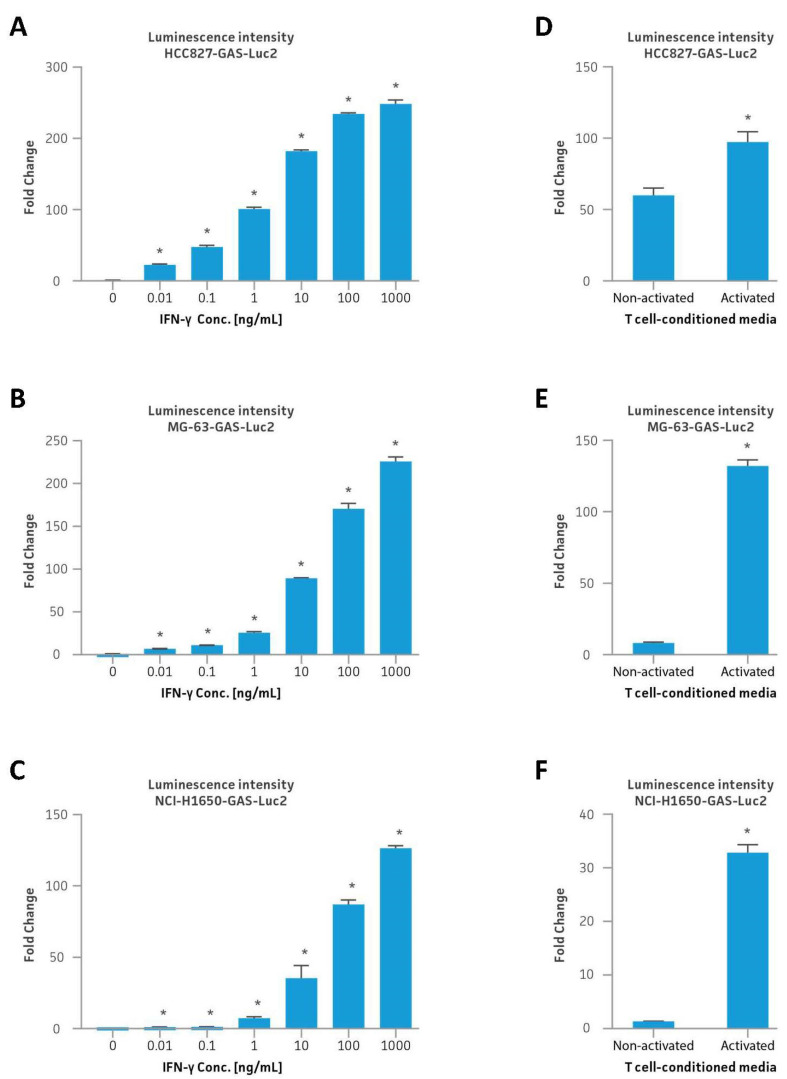
Cytokine stimulation of monoclonal GAS-Luc2 cell lines with IFNγ or CD8+ cytotoxic T cell-conditioned media. (**A**–**C**) The luminescence intensity of HCC827-GAS-Luc2 (**A**), MG-63-GAS-Luc2 (**B**), or NCI-H1650-GAS-Luc2 (**C**) cell line upon IFNγ stimulation (0.01–1000 ng/mL). (**D**–**F**) The luminescence intensity of HCC827-GAS-Luc2 (**D**), MG-63-GAS-Luc2 (**E**), or NCI-H1650-GAS-Luc2 (**F**) cell line upon stimulation with conditioned media collected from non-activated or activated CD8+ cytotoxic T cells. Error bars indicate standard deviation (SD). N = 3 in all experiments. * *p* < 0.05.

Following single-cell clonal expansion, the luminescence signal intensity upon IFNγ stimulation was re-evaluated for the selected monoclonal HCC827-GAS-Luc2, MG-63-GAS-Luc2, and NCI-H1650-GAS-Luc2 cell lines with IFNγ concentrations ranging 0.01–1000 ng/mL (Figure 2A–C). Initial stimulation tests of the monoclonal HCC827-GAS-Luc2 cell line demonstrated the highest sensitivity to IFNγ administration as compared to the other two cell lines, exhibiting an average 15-fold increase in RLU values relative to untreated controls following 0.01 ng/mL IFNγ administration (Figure 2A). Increased luminescence was observed with increasing IFNγ administration, with 1000 ng/mL IFNγ administration resulting in a 250-fold increase in RLU values as compared to untreated controls. Similarly, the selected monoclonal MG-63-GAS-Luc2 and NCI-H1650-GAS-Luc2 cell lines also exhibited a robust and dose-dependent luminescence signal to IFNγ administration, demonstrating a 230-fold and 130-fold increase, respectively, in RLUs relative to untreated controls upon 1000 ng/mL IFNγ administration (Figure 2B,C).

Following the IFNγ stimulation assays, signaling activation of luciferase expression from the engineered cell line was also assessed using conditioned media from primary human CD8+ T cells (Figure 2D–F). Here, the GAS-Luc2-engineered cell lines were treated for 24 h with conditioned media from either non-activated or CD3/CD28-bead-activated primary human CD8+ T cells, followed by luminescence assessment. The activated T cell–conditioned media stimulation in HCC827-GAS-Luc2, MG-63-GAS-Luc2, and NCI-H1650-GAS-Luc2 cell lines led to a 100-fold, 130-fold, and 30-fold increase in RLUs compared to untreated controls, respectively. In contrast, non-activated T cell-conditioned media stimulation resulted in a 50-fold increase in RLUs in HCC827-GAS-Luc2, which previously showed the highest sensitivity to the lowest dose of IFNγ, and more muted responses in the MG-63-GAS-Luc2 and NCI-H1650-GAS-Luc2 cell lines.

### 3.6. Application in the PD-L1 Immune Checkpoint Inhibitor Platform via Co-Culture with Primary Human CD8+ Cytotoxic T Cells

**Figure 3:**
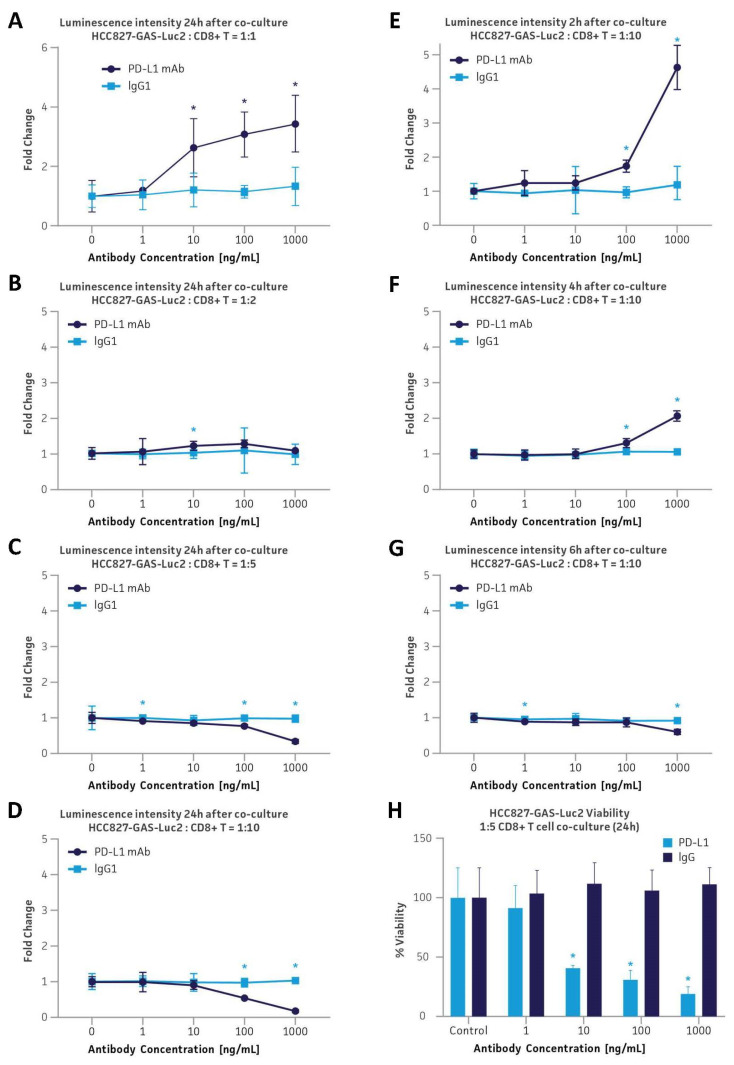
Co-culture of monoclonal HCC827-GAS-Luc2 with primary human CD8+ cytotoxic T cells at varying cell ratios and co-culture durations in the presence of a PD-L1 blocking antibody. (**A**–**D**) The luminescence intensity from HCC827-GAS-Luc2 cells after 24-h co-culture with CD8+ cytotoxic T cells at a 1:1 (**A**), 1:2 (**B**), 1:5 (**C**), and 1:10 (**D**) ratio of target cells to effector cells. (**E**–**G**) The luminescence intensity from HCC827-GAS-Luc2 cells after co-culture at a 1:10 ratio with CD8+ cytotoxic T cells for periods of 2€(**E**), 4 h (**F**), and 6 h (**G**). (**H**) The viability of HCC827-GAS-Luc2 after 24 h of co-culture with a 1:5 ratio with CD8+ cytotoxic T cells for 24 h. During the co-culture, the cells were administered with either PD-L1 mAb or isotype control IgG (1–1000 ng/mL). Error bars indicate standard deviation (SD). N = 3 in all experiments. * *p* < 0.05.

The ability of GAS-Luc2 cell lines to serve as reporter cell lines for immune checkpoint drug screening in solid tumors was assessed via co-culture with primary human immune cells, including CD8+ cytotoxic T cells, CD4+ helper T cells, and CD56+ NK cells. First, monoclonal HCC827-GAS-Luc2 cells were co-cultured with different ratios of CD8+ cytotoxic T cells under varying co-culturing times. Cells were also administered various concentrations of either a PD-L1 blocking monoclonal antibody (mAb) or isotype control IgG (1–1000 ng/mL). PD-L1 mAb administration served as a blocker of the checkpoint inhibitor, preventing the cancer cells from silencing the immune activity of CD8+ cytotoxic T cells via their PD-L1 to PD-1 binding. Increasing the PD-L1 mAb concentrations in the co-culture models resulted in a dose-dependent increase in luciferase expression of the HCC827-GAS-Luc2 cell line, exhibiting a 3- and 5-fold increase of luminescence intensity relative to untreated controls after 24- and 2-h co-culture incubation periods with 1:1 and 1:10 ratios of HCC827-GAS-Luc2 to CD8+ T cells, respectively (Figure 3A,E). In contrast, increasing isotype control IgG concentrations failed to induce any change in luciferase expression from HCC827-GAS-Luc2 cancer reporter cell lines relative to untreated controls.

To further demonstrate the ability of the reporter cell line to mimic the in vivo CD8+ cytotoxic T cell-mediated cancer cell killing, additional co-culture conditions were evaluated using HCC827-GAS-Luc2 cells with increased CD8+ T cell populations, as well as increased co-culture incubation times. Both conditions were found to greatly affect maximum luciferase expression. Increasing the ratio of CD8+ T cells to reporter cells proportionately decreased luminescence intensity after 24 h of co-culture, with 1:5 and 1:10 HCC827-GAS-Luc2:CD8+ T cell ratios exhibiting no detectable luminescence signaling under 1000 ng/mL PD-L1 mAb administration (Figure 3A–D). Similarly, increasing the co-culturing incubation period was found to correlatedly decrease the luciferase expression when the ratio of effector cells to target cells remained consistently high (1:10 ratio of HCC827-GAS-Luc2 to CD8+ T cells). Here, 2-, 4-, 6-, and 24-h co-culture incubation with 1000 ng/mL PD-L1 mAb administration demonstrated a 5-, 1-, 0.7-, and 0-fold increase in luminescence intensity relative to untreated controls, respectively (Figure 3D–G). These phenomena were attributed to the cell death of HCC827-GAS-Luc2 cells, killed by CD8+ cytotoxic T cells over time or via elevated cytotoxicity with an increased number of CD8+ cytotoxic T cells added to the culture. In fact, a simple cell viability study revealed that cancer cell viability decreases significantly during the co-culture with CD8+ cytotoxic T cells over time (Figure 3H). The cell-killing effect that a 24-h co-culture of HCC827-GAS-Luc2 with CD8+ cytotoxic T cells at a 1:5 ratio of cancer cells to effector cells led to approximately 40%, 31%, and 19% cancer cell viability with the administration of 10, 100, and 1000 ng/mL PD-L1 mAb, respectively. While the system was shown to effectively mimic the in vivo cancer cell killing by CD8+ cytotoxic T cells, the luminescence signal from the cancer cells naturally reached its maximum intensity before they were killed. Therefore, when designing cell-based assays using luciferase reporter systems, it is critical to consider the effect of cytotoxicity on the bioluminescence signal and optimize the assay in terms of co-culturing duration and target to effector cell ratio.

### 3.7. Application in the PD-L1 & TIGIT Immune Checkpoint Inhibitor Platforms via Co-Culture with Primary Human CD4+ Helper T Cells

**Figure 4:**
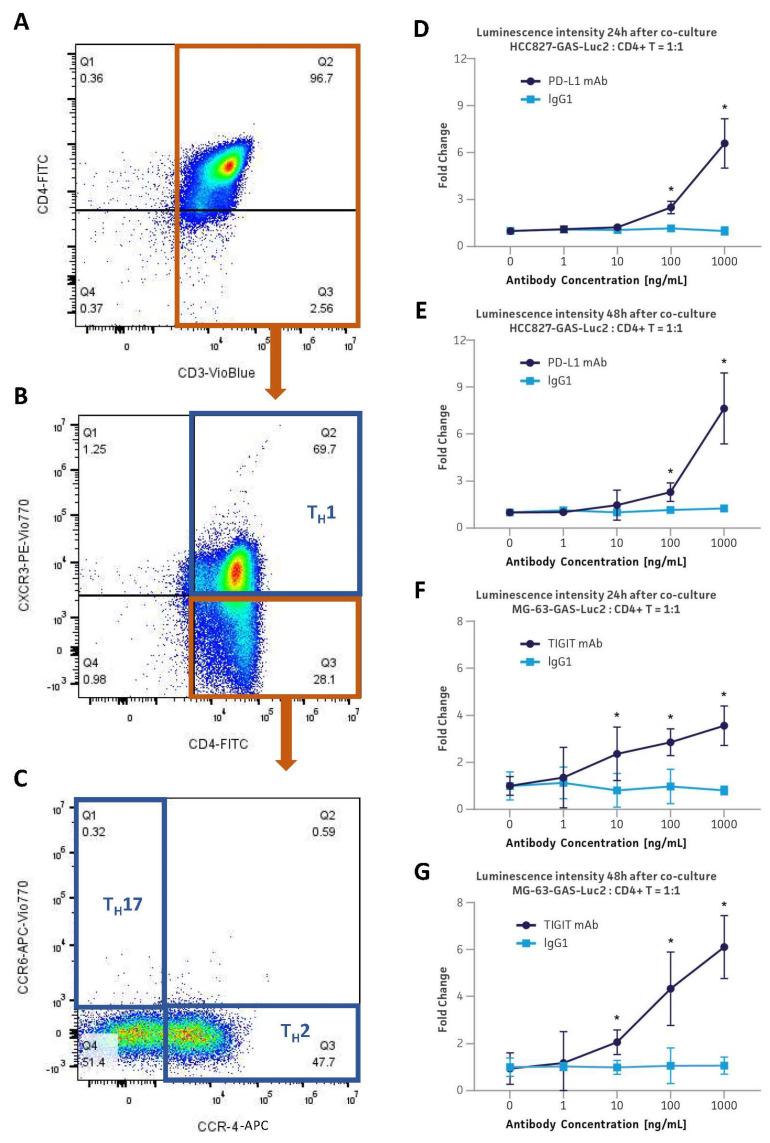
Co-culture of monoclonal HCC827-GAS-Luc2 or MG-63-GAS-Luc2 cells with primary human CD4+ helper T cells in the presence of a respective blocking antibody after CD4+ helper T cell subset phenotyping. (**A**–**C**) The flow cytometry analysis of the helper T cell subsets T_H_1, T_H_17, and T_H_2. (**D**,**E**) The luminescence intensity from HCC827-GAS-Luc2 cells after co-culture at a 1:1 ratio with CD4+ helper T cells for 24 h (**D**) or 48 h (**E**) in the presence of a PD-L1 mAb or isotype control IgG1 (1–1000 ng/mL). (**F**,**G**) The luminescence intensity from MG-63-GAS-Luc2 cells after co-culture at a 1:1 ratio with CD4+ helper T cells for 24 h (**F**) or 48 h (**G**) in the presence of a TIGIT mAb or isotype control IgG (1–1000 ng/mL). Error bars indicate standard deviation (SD). N = 3 in all experiments. * *p* < 0.05.

Since the GAS-Luc2 reporter cell lines were designed to respond to JAK/STAT signaling pathway activation with luciferase expression, they have the versatility to work with any type of immune cells that secrete the signaling-activating cytokines, such as IFNγ. Of the three major effector CD4+ helper T subsets, Th1 is characterized by IFNγ secretion. To ensure that the primary human CD4+ helper T cells for the co-culture assay include the IFNγ releasing population, a subset phenotyping was conducted by flow cytometry using anti-CD3, anti-CD4, anti-CXCR3, anti-CCR4, and anti-CCR6 antibodies (Figure 4A–C). The result showed that approximately 70% of the CD4+ population was also CXCR3+, which is the characteristic cell surface marker for Th1. 47.7% of the CD4+/CXCR3- population was CCR4+/CCR6-, indicating 13.4% of the whole CD4+ population was Th2. Meanwhile, only 0.32% of the CD4+/CXCR3- population was CCR4-/CCR6+, depicting a negligible size of population of Th17 and therefore leaving the majority of the CD4+/CXCR3- population to be negative for both CCR4 and CCR6.

Upon confirming the preponderance of IFNγ-secreting Th1 population among the CD4+ helper T cells, these cells were co-cultured with HCC827-GAS-Luc2 cells or MG-63-GAS-Luc2 cells for 24 h or 48 h at a 1:1 cell ratio (Figure 4D–G). Both HCC827-GAS-Luc2 cells and MG-63-GAS-Luc2 cells demonstrated a dose-dependent increase in luminescence intensity with the administration of the PD-L1 blocking mAb and TIGIT blocking mAb, respectively. TIGIT mAb was used to inhibit the binding of the immune checkpoint molecule TIGIT to CD155, the ligand of TIGIT and the immune checkpoint marker of MG-63-GAS-Luc2. For HCC827-GAS-Luc2, a 6- and 8-fold increase in luminescence intensity at 24 and 48 h of co-culture, respectively, relative to the IgG control with the addition of 1000 ng/mL PD-L1 mAb was observed. Similarly, MG-63-GAS-Luc2 cells exhibited a 3- and 6-fold increase in luminescence intensity under the conditions of 24 and 48 h of co-culture, respectively; with 1000 ng/mL TIGIT mAb administration. These results show that the antibody-mediated immune checkpoint blockade activates the CD4+ helper T cells to release pro-inflammatory cytokines, such as IFNγ, which subsequently activate the JAK/STAT signaling pathway in the GAS-Luc2 engineered cells, leading to the luciferase expression from the reporter cells.

### 3.8. Application in the PD-L1 Immune Checkpoint Inhibitor Platform via Co-Culture with Primary Human CD4+ Helper T Cells by Employing an Anti-CD3/CD28-Expressing aAPC

**Figure 5:**
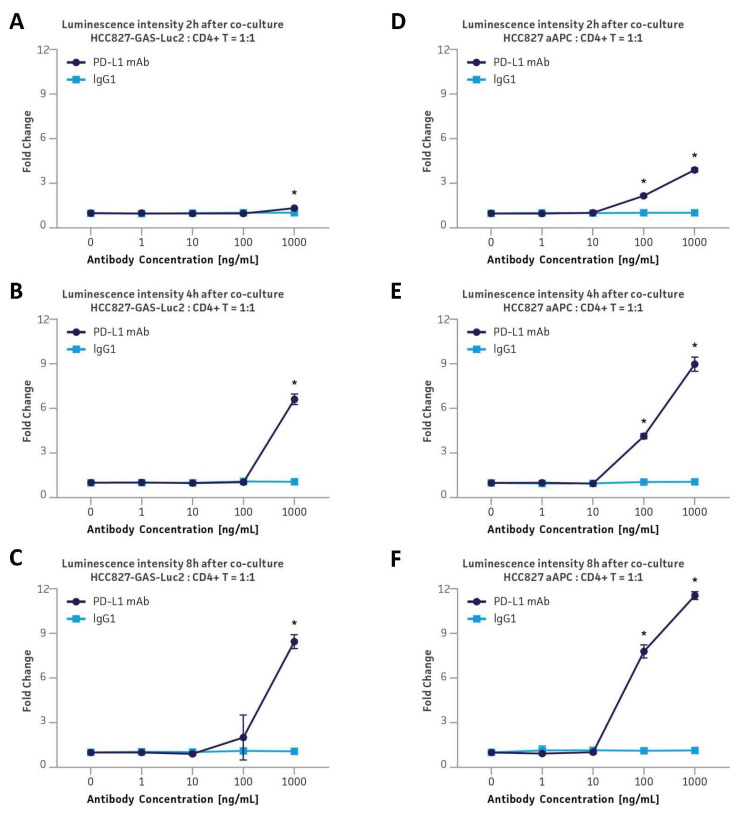
Comparison of HCC827-GAS-Luc2 and HCC827-GAS-Luc2-aAPC in co-culture with primary human CD4+ helper T cells in the presence of a PD-L1 blocking antibody. (**A**–**C**) The luminescence intensity from HCC827-GAS-Luc2 cells after co-culture at a 1:1 ratio with CD4+ helper T cells for 2 h (**A**), 4 h (**B**), or 8 h (**C**). (**D**–**F**) The luminescence intensity from HCC827-GAS-Luc2-aAPC cells after co-culture at a 1:1 ratio with CD4+ helper T cells for 2 h (**D**), 4 h (**E**), or 8 h (**F**). During the co-culture, the cells were administered with either PD-L1 mAb or isotype control IgG (1–1000 ng/mL). Error bars indicate standard deviation (SD). N = 3 in all experiments. * *p* < 0.05.

Immune checkpoint blockade-mediated T cell activation is normally preceded by the TCR recognition of the peptide-loaded MHC molecules on the APCs. However, artificially designed APCs that express anti-CD3 and anti-CD28 antibodies on the cell surface can bypass the antigen recognition and swiftly initiate the TCR signaling. To enhance the reporter function of the GAS-Luc2 engineered cell lines by expressing anti-CD3 and anti-CD28 receptors, the reporter cell line HCC827-GAS-Luc2 was lentivirally transduced with the two chimeric stimulatory receptors (CSRs): anti-CD3 scFv-GFP and anti-CD28 scFV-mCherry [20,21]. Subsequent bulk sorting for GFP+/mCherry+ cells yielded a multi-clone aAPC pool (Supplemental Figure S7). The HCC827-GAS-Luc2-aAPC cells were then co-cultured with CD4+ helper T cells at a 1:1 ratio of target to effector cells for 2 h (Figure 5D), 4 h (Figure 5E), or 8 h (Figure 5F) in the presence of either PD-L1 blocking mAb or isotype control IgG (1–1000 ng/mL). To compare the results to the non-aAPC cells, HCC827-GAS-Luc2 cells were co-cultured with CD4+ helper T cells under the same conditions (Figure 5A–C). The aAPC cells were observed to exhibit brighter luminescence signal more quickly and with a lower concentration of the blocking antibody as compared to the signal from the non-aAPC counterpart. For example, with the administration of 1000 ng/mL PD-L1 mAb, the luminescence signal intensity from the aAPC cells increased 4-fold after 2 h, 9-fold after 4 h, and 12-fold after 8 h of the co-culture. Even with 100 ng/mL PD-L1 mAb, the signal still increased 2-fold, 4-fold, and 8-fold at 2 h, 4 h, and 8 h, respectively. In contrast, the luminescence intensity from the non-aAPC cells with the administration of 1000 ng/mL PD-L1 mAb increased 1.3-fold, 6-fold, and 8-fold after 2 h, 4 h, and 8 h of co-culture, respectively. With 100 ng/mL PD-L1 mAb, a statistically significant signal increase was not observed in any of the above co-culture conditions.

### 3.9. Application in the B7-H3 Immune Checkpoint ADCC Platform via Co-Culture with Primary Human CD56+ NK Cells

**Figure 6:**
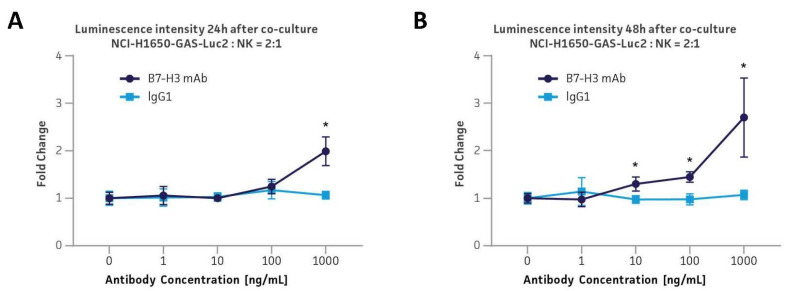
Co-culture of monoclonal NCI-H1650-GAS-Luc2 with primary human CD56+ NK cells in the presence of a B7-H3 ADCC antibody. (**A**,**B**) The luminescence intensity from NCI-H1650-GAS-Luc2 after co-culture with CD56+ NK cells at a 2:1 ratio of target to effector cells for 24 h (**A**) or 48 h (**B**). During the co-culture, the cells were administered with either B7-H3 mAb or isotype control IgG (1–1000 ng/mL). Error bars indicate standard deviation (SD). N = 3 in all experiments. * *p* < 0.05.

As NK cells are another primary source of IFNγ and play a significant role in the immune response, the reporter function of the GAS-Luc2 cells was further examined by the co-culture assay with the primary human CD56+ NK cells. NCI-H1650-GAS-Luc2 cells were co-cultured with CD56+ NK cells at a 2:1 ratio of target cells to effector cells with the administration of either a B7-H3 ADCC mAb or isotype control IgG (Figure 6A,B). B7-H3-targeted ADCC mAb functions by linking NK cells to B7-H3-expressing tumor cells that are subject to increased IFNγ paracrine signaling from the NK cells. The B7-H3 mAb–treated NCI-H1650-GAS-Luc2 cells demonstrated a dose-dependent increase in the luminescence intensity relative to untreated controls. For instance, 1000 ng/mL B7-H3 mAb elicited a 2- and 3-fold increase in luminescence intensity relative to untreated controls under the conditions of 24 and 48 h of co-culture, respectively. On the other hand, increasing isotype control IgG concentrations did not increase the luciferase expression in NCI-H1650-GAS-Luc2 cells. Overall, the data show that the GAS-Luc2 cells can effectively assess the efficacy of immune checkpoint-targeting antibodies in activating the immune cells in various co-culture conditions. Moreover, these results indicate that the application of these reporter cells is not limited to the co-culture studies with CD8+ cytotoxic T cells but can also be used in diverse co-culture studies that incorporate other types of IFNγ-releasing immune cells such as CD4+ helper T cells (Th1) and CD56+ NK cells for various immunomodulatory drug screening.

## 4. Discussion

The vast, and most popular, open-to-the-public cell line gene expression database of The Cancer Cell Line Encyclopedia (CCLE) developed by the Broad Institute^®^ (Cambridge, MA, USA) is largely based on mRNA expression [22]. The current phase III of the CCLE project is to characterize gene expression at the protein level by mass spectrometry. Such a tremendous effort is now under active development [23]. In parallel, drug discovery and development in the immuno-oncology field has sped up dramatically in recent years, with 2030 agents and 265 targets in 2017, and 4720 agents and 504 targets in 2020 globally, including immune checkpoint inhibitor, ADCC, and CAR-T approaches [24]. To facilitate such a speedy expansion in the immuno-oncology field, ATCC^®^ conducted a comprehensive protein profiling of common immune checkpoint molecules and their paired ligands in over 50 tumor cell lines and over 10 immune cell lines internally. Such a protein profiling was performed through the technique of flow cytometry by employing all well-characterized antibodies in the literature (Supplemental Figure S1). The significance of this protein profiling data is that it is complementary to the gene expression database of the CCLE, which has been widely used in cancer research [25,26]. Although tremendously useful, both the phase I and phase II of the CCLE gene expression database are mRNA expression datasets instead of protein expression [22,27,28]. Studies have confirmed that there is a clear discrepancy of gene expression at the mRNA level and protein level, although they are certainly positively correlated, and mRNA expression is far from perfect in predicting protein expression levels [29,30].

Based on our comprehensive proteomics data, we selected and engineered three solid tumor cell lines to generate robust in vitro immune checkpoint reporting systems. The HCC827, MG-63, and NCI-H1650 cell lines—each with high natural expression of immune checkpoint proteins PD-L1, CD155, and B7-H3, respectively—were developed into an IFNγ-IFNγR JAK-STAT GAS-Luc2 reporter systems that are capable of reliably reporting the activity of IFNγ from activated primary human immune cells that mimic the in vivo cancer cell killing events. Compared to other immune checkpoint assays on the market that use engineered cancer cell lines with ectopic overexpression of certain immune checkpoint molecules, these novel immune checkpoint assay reporting systems are much more physiologically relevant, as the cancer cells that we selected have naturally high expression of relevant immune checkpoint molecules [6].

To maximize assay versatility, we incorporated the IFNγ-IFNγR JAK-STAT GAS-Luc2 reporting system in the cancer cells so users can employ multiple kinds of primary human immune cells for the counterpart of the cancer cells in the full human co-culture assay. For example, we can use T cells that were newly defined by single cell sequencing (scRNAseq) [31], CAR-T cells [32], B cells [33], and myeloid cells [34]. Additionally, these cells could be used in combination with or without other human tumor microenvironmental cells, such as primary human fibroblasts [35] and endothelial cells [36], for better modeling of the in vivo T cell-mediated killing of cancer events in the solid tumor microenvironment. All these permutations are improvements over other immune checkpoint assays on the market whose signaling reporting system is restricted to a non-cytotoxic T cell line instead of primary human immune cells, such as the CD4+ Jurkat T cell line with an NFAT-Luc2 reporter [6].

Recent studies have demonstrated the importance of IFNγ-IFNγR JAK-STAT signaling for T cell-mediated killing of cancer cells specifically in solid tumors but not in liquid cancers [15]. Additional research has unveiled that PD-L1 blockade rejuvenates CAR-T cell function by modulating IFNγ to impact CD163+ M2 macrophages [37]. Another study recently reported that the IFNγ from CD4+ T cells in the solid tumor microenvironment drives microglial phagocytosis of glioblastoma cells [16]. These studies suggest that IFNγ plays a critical role in orchestrating the collaborative synergy among different immune cells, leading to effective cytotoxicity against cancer cells within the solid tumor microenvironment. Our fully human cancer GAS-Luc2 reporting system better supports the immune checkpoint drug discovery and development in solid tumors where the success rate of immune checkpoint blockade therapy has been markedly lower. Therefore, because of its high endogenous target marker expression, robust bioluminescent signal, and assay versatility, the GAS-Luc2 reporter cell lines are ideal for assaying and screening candidate PD-1/PD-L1, CD155/TIGIT, and B7-H3 immune checkpoint inhibitors and ADCC antibody agents.

One distinctive challenge of using a reporter cell line is its allogeneic nature when incorporated into a co-culture assay with primary immune cells [38]. Immune checkpoint blockade-mediated T cell activation in vivo is initiated by recognition of peptide-loaded MHCs on antigen-presenting cells by T cell receptors (TCRs). However, fulfilling this TCR recognition step ex vivo is especially challenging because finding matching donors of the established cell lines to primary immune cells is impractical. Multiple recent studies have reported that artificially engineered expression of CD3- and CD28-activating antibodies on the cell surface can circumvent the initial TCR recognition step and trigger the TCR signaling for T cell activation ex vivo [20,21,39]. In fact, our T cell co-culture experiments with anti-CD3/CD28-expressing HCC827-GAS-Luc2-aAPC elicited more rapid and elevated responses from the aAPC cells compared to the non-aAPC cells. These results highlight the further potential of the reporter cell lines and their broad applications in cancer immunotherapy drug screening.

## 5. Conclusions

In conclusion, recent research underscores the pivotal role of the interferon gamma receptor (IFNγR) pathway in orchestrating T cell-mediated destruction of solid tumors. Through both direct induction of tumor cell death and indirect stimulation of myeloid phagocytosis in the tumor microenvironment, interferon gamma (IFNγ) emerges as a key player in bolstering cytotoxicity against solid tumors. Despite these advancements, the complexities of human ex vivo immune checkpoint drug screening persist as a formidable challenge. Our study introduces an innovative approach utilizing the engineered gamma interferon activation site response element luciferase reporter (GAS-Luc2) for comprehensive screening in varied ex vivo T cell–solid tumor cell co-culture systems. By profiling cell surface proteins and engineering reporter tumor cell lines expressing key immune checkpoints, we enable precise evaluation of checkpoint inhibitor efficacy through quantifiable bioluminescent signals. Furthermore, our findings extend to the detection of paracrine IFNγ signaling in ADCC drug screening and the development of an artificial antigen-presenting cell line (aAPC), enhancing T cell responsiveness and offering promising avenues for advancing various immune checkpoint drug screening methodologies.

## Data Availability

The data presented in this study are available on request from the corresponding author.

## Supplementary Materials

The following supporting information can be downloaded at: https://www.mdpi.com/article/10.3390/cancers16111965/s1, Figure S1: List of antibodies used for flow cytometry; Figure S2: Representative histograms assessing expression levels of immune checkpoint molecule ligands; Figure S3: Representative histograms assessing expression levels of immune checkpoint molecules; Figure S4: Luminescence intensity evaluation of GAS-Luc2 engineered candidate cell lines; Figure S5: Comparison of immune checkpoint molecule ligand expression between parental and GAS-Luc2 engineered cell lines; Figure S6: Comparison of cell morphology between parental and GAS-Luc2 engineered cell lines; Figure S7: Flow cytometry analysis of HCC827-GAS-Luc2-aAPC multi-clone pool.
