# Supplementary material for: GAS-Luc2 Reporter Cell Lines for Immune Checkpoint Drug Screening in Solid Tumors
Source: Cancers (Basel). 2024 May 22;16(11):1965. doi: 10.3390/cancers16111965 (PMC11171215; doi:10.3390/cancers16111965)
Supplement: Supplementary file 1 [file cancers-16-01965-s001.zip › cancers-3001013-supplementary.pdf]

<u>Supplemental Data</u>

GAS-Luc2 Reporter Cell Lines for Immune Checkpoint Drug Screening in Solid Tumors

Hyeyoun Chang, John G. Foulke, Luping Chen, Fang Tian * and Zhizhan Gu *

American Type Culture Collection (ATCC), Manassas, VA 20110, USA

* Correspondence: zgu@atcc.org (Z.G.); ftian@atcc.org (F.T.)

List of antibodies used for flow cytometry

Target	Fluorophore	Vendor	Reference #
HLA Class I	PE	BD Pharmingen	555553
HLA Class II	FITC	BD Pharmingen	555558
PD-L1	APC	BD Pharmingen	563741
PD-L1	PE	BD Pharmingen	557924
PD-L2	PerCP-Cy5.5	BD Pharmingen	564256
B7-H3	APC	Miltenyi Biotec	130-095-522
B7-H4	APC	BD Pharmingen	562787
HVEM	FITC	Miltenyi Biotec	130-101-595
4-1BBL	PE	BD Pharmingen	559446
ICOS-L	FITC	Miltenyi Biotec	130-124-242
CD155	PE	BD Pharmingen	566718
CD80	PE	BD Pharmingen	566992
CD86	FITC	BD Pharmingen	555657
PD-1	APC	BD Pharmingen	558694
CTLA-4	APC	BD Pharmingen	555855
CTLA-4	PE	BD Pharmingen	555853
LAG-3	PE	BD Pharmingen	565616
TIM-3	PE	BD Pharmingen	563422
BTLA	PerCP	R&D Systems	FAB3354C
VISTA	PE	BD Pharmingen	566669
TIGIT	FITC	Invitrogen	11-9500-42
4-1BB	APC	BD Pharmingen	550890
ICOS	PE	BD Pharmingen	557802
CD30	APC	BD Pharmingen	563500
CD28	FITC	BD Pharmingen	555728
CD28	PE	BD Pharmingen	555729
OX40	FITC	BD Pharmingen	555837
GITR	APC	Miltenyi Biotec	130-118-977
CD226	FITC	BD Pharmingen	559788
CD4	FITC	BD Pharmingen	561005
CD8	APC	Miltenyi Biotec	130-110-679
CD8	FITC	BD Pharmingen	561947
CD3	VioBlue	Miltenyi Biotec	130-113-695
CXCR3	PE-Vio770	Miltenyi Biotec	130-120-593
CCR4	APC	Miltenyi Biotec	130-117-525
CCR6	APC-Vio770	Miltenyi Biotec	130-125-953

Supplemental Fig. S1. List of antibodies used for flow cytometry.

Supplemental Figure S2

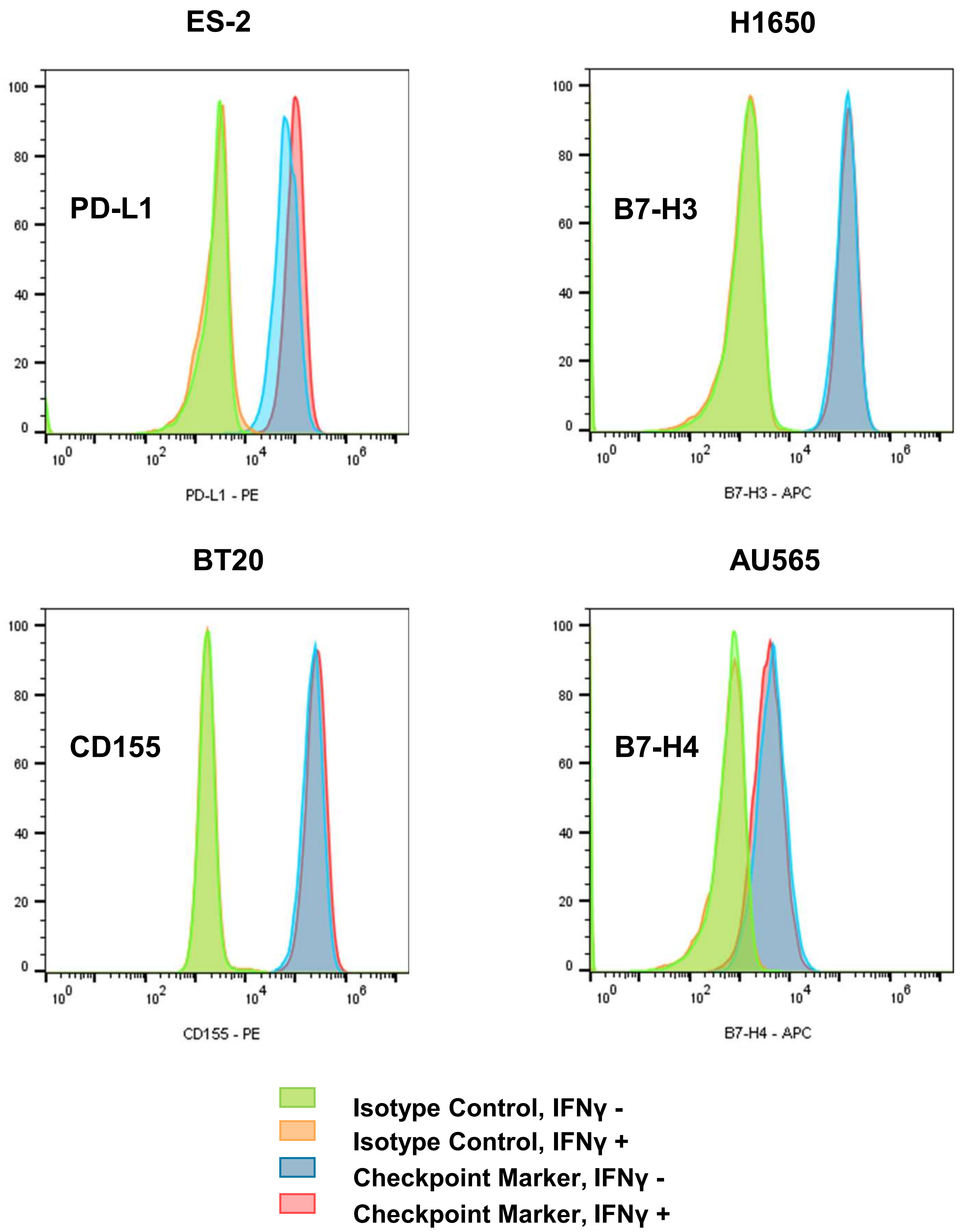

Supplemental Fig. S2. Representative histograms assessing expression levels of immune checkpoint molecule ligands. Flow cytometry analysis of candidate cancer cell lines and their representative expression of immune checkpoint molecule ligands with relevant controls. Experiments have been repeated three times and the representative results are shown.

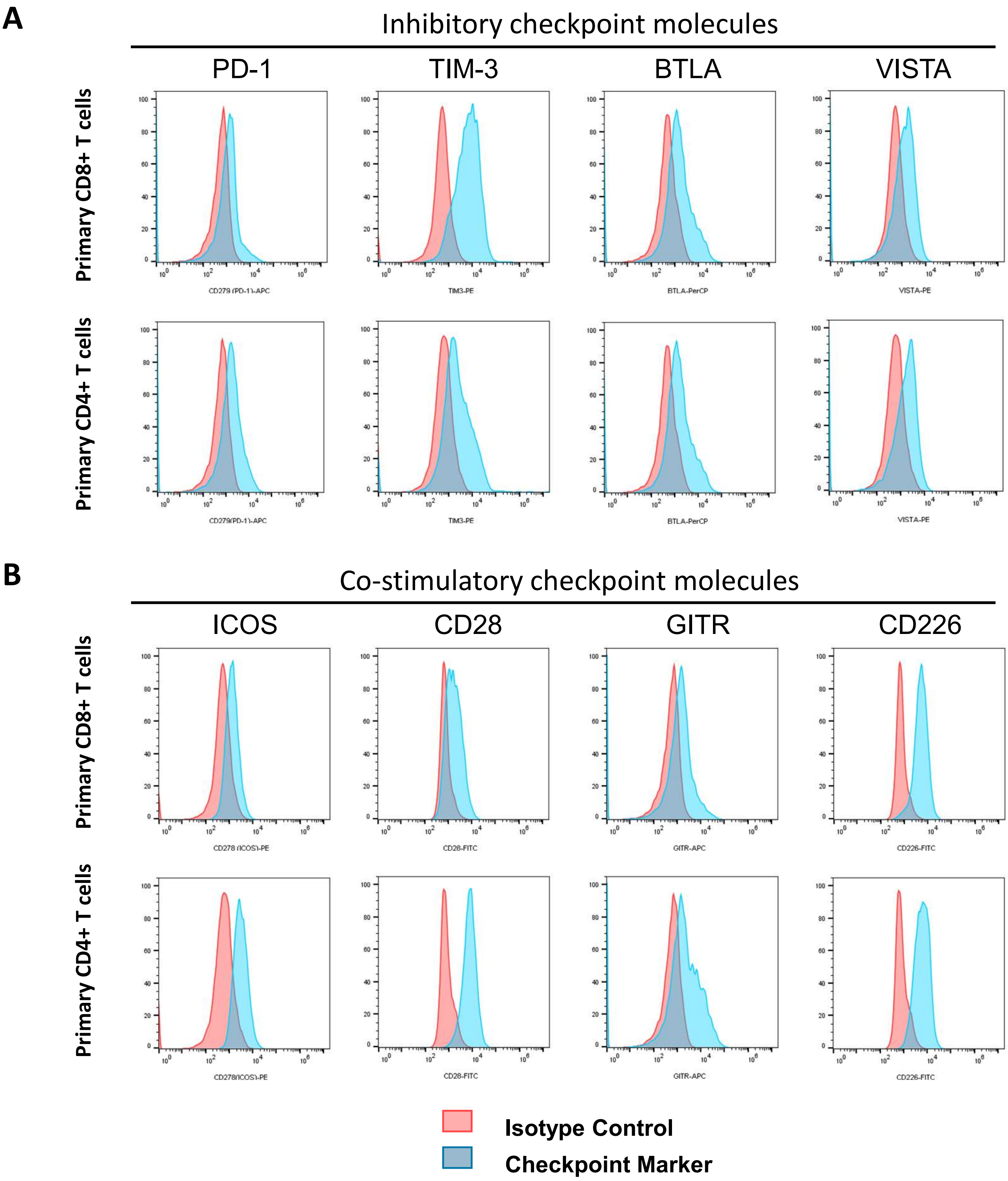

Supplemental Fig. S3. Representative histograms assessing expression levels of immune checkpoint molecules. Flow cytometry analysis of primary human CD8+ and CD4+ T cells and their representative expression of immune checkpoint molecules with relevant controls. Experiments have been repeated three times and the representative results are shown.

Supplemental Figure S4

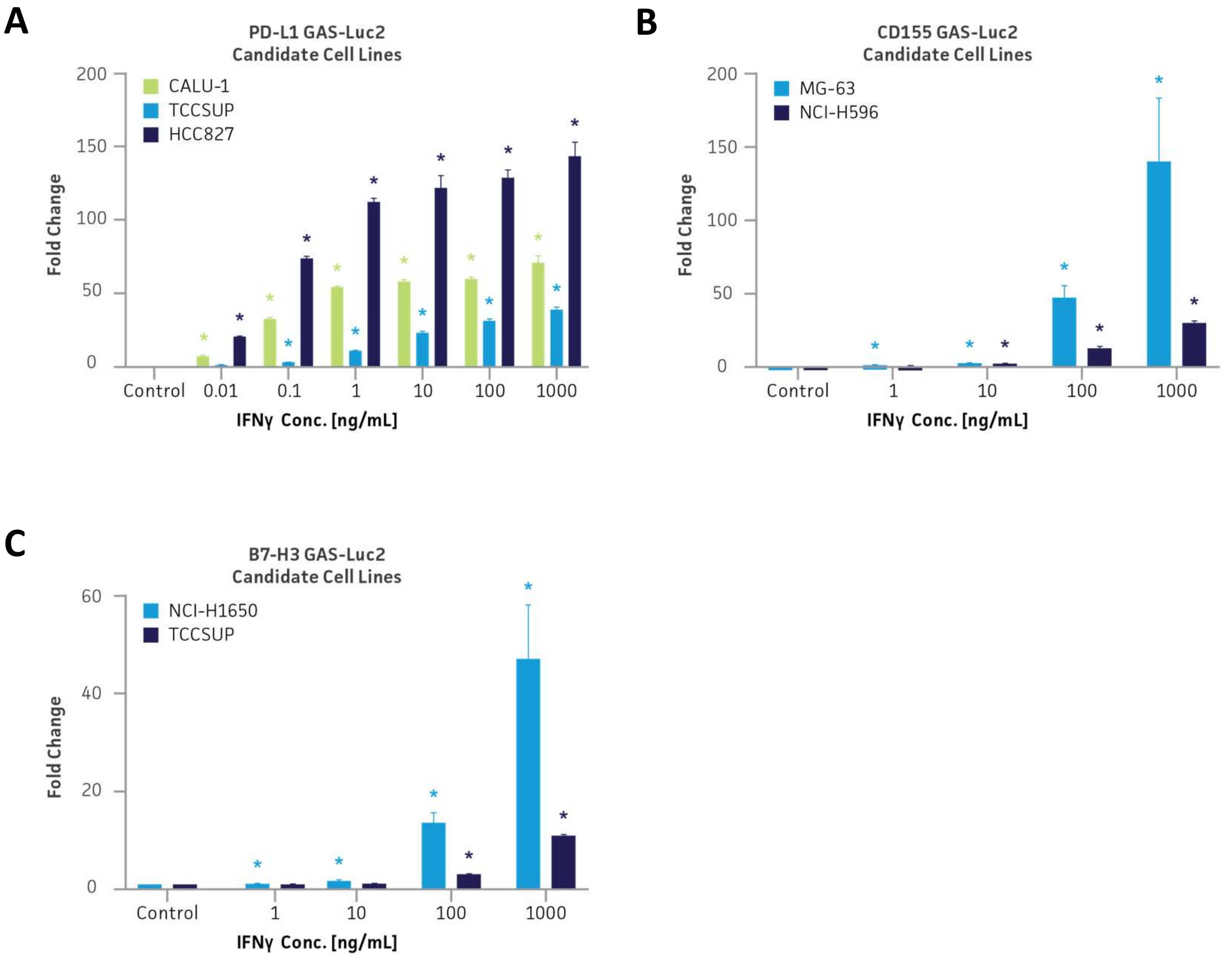

Supplemental Fig. S4. Luminescence intensity evaluation of GAS-Luc2 engineered candidate cell lines. Candidate cell lines were selected for GAS-Luc2 modification based on high expression of selected immune checkpoint markers and were assessed via IFN-γ cytokine stimulation assay following viral transduction. Antibiotic-selected GAS-Luc2 engineered multi-clone cells that endogenously expressed high PD-L1 (A), CD155 (B), or B7-H3 (C) were administered IFN-γ of different concentrations (1-1,000 ng/ mL). Error bars indicate standard deviation (SD). N=3 in all experiments. *$P < 0.05$.

Supplemental Figure S5

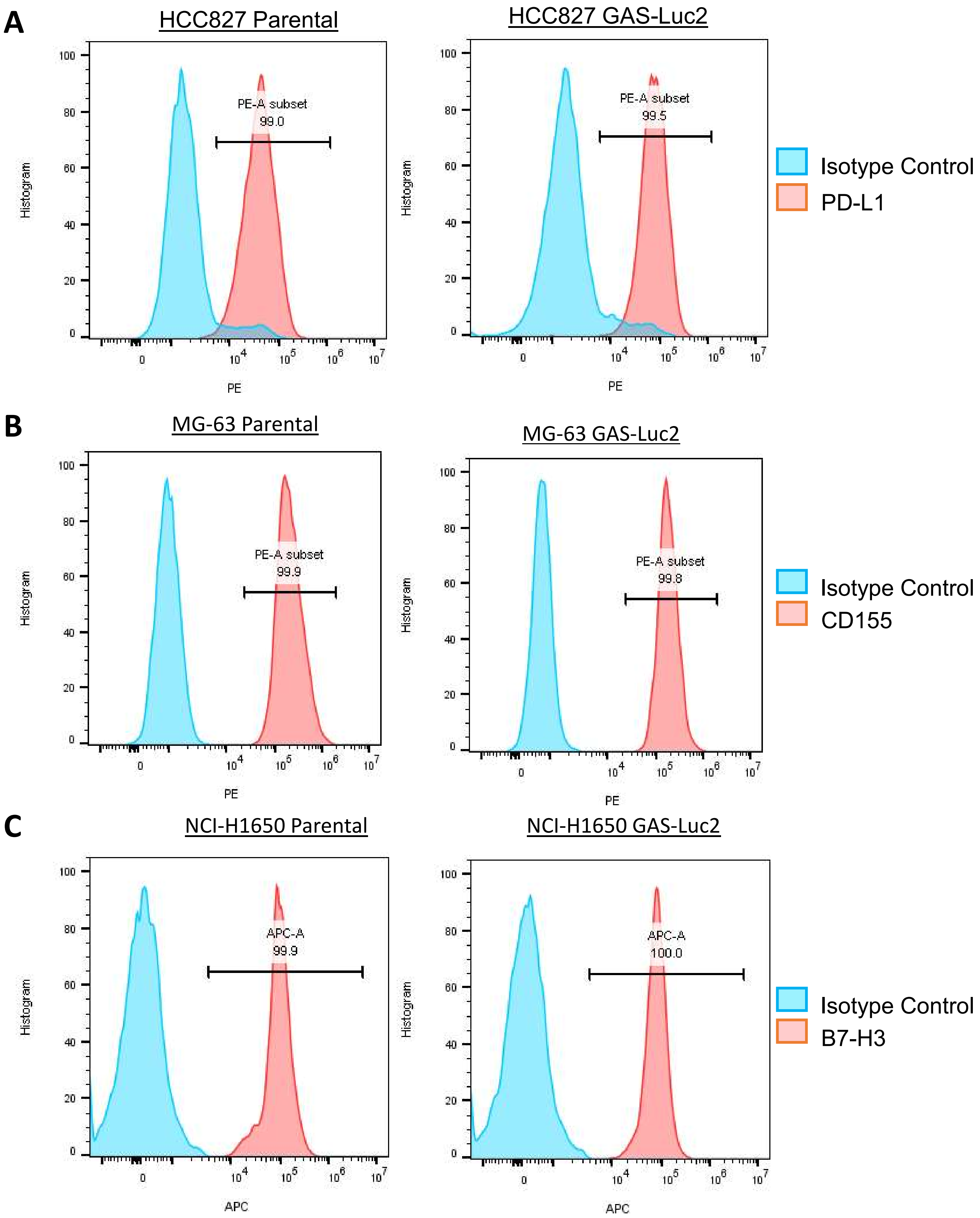

Supplemental Fig. S5. Comparison of immune checkpoint molecule ligand expression between parental and GAS-Luc2 engineered cell lines. Flow cytometry data on the expression level of PD-L1 (A), CD155 (B), and B7-H3 (C) in HCC827, MG-63, and NCI-H1650 parental and GAS-Luc2 engineered monoclonal cell lines, respectively. Experiments have been repeated three times and the representative results are shown.

Supplemental Figure S6

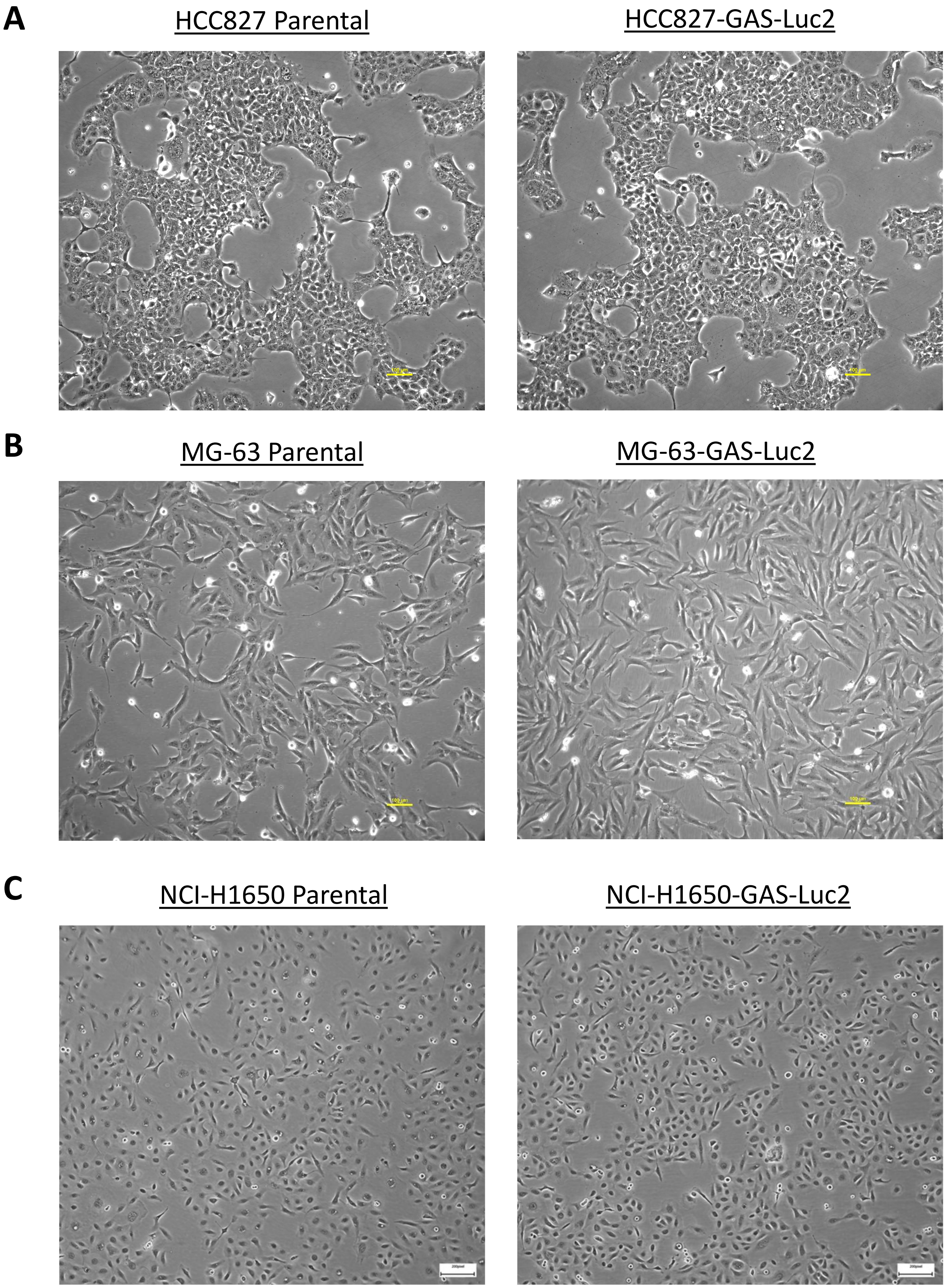

Supplemental Fig. S6. Comparison of cell morphology between parental and GAS-Luc2 engineered cell lines. (A) HCC827 parental and HCC827-GAS-Luc2 cells, (B) MG-63 parental and MG-63-GAS-Luc2 cells, and (C) NCI-H1650 parental and NCI-H1650-GAS-Luc2 cells. Images taken by Eclipse TE300 inverted microscope (Nikon). Size bar = 100 µm.

Supplemental Figure S7

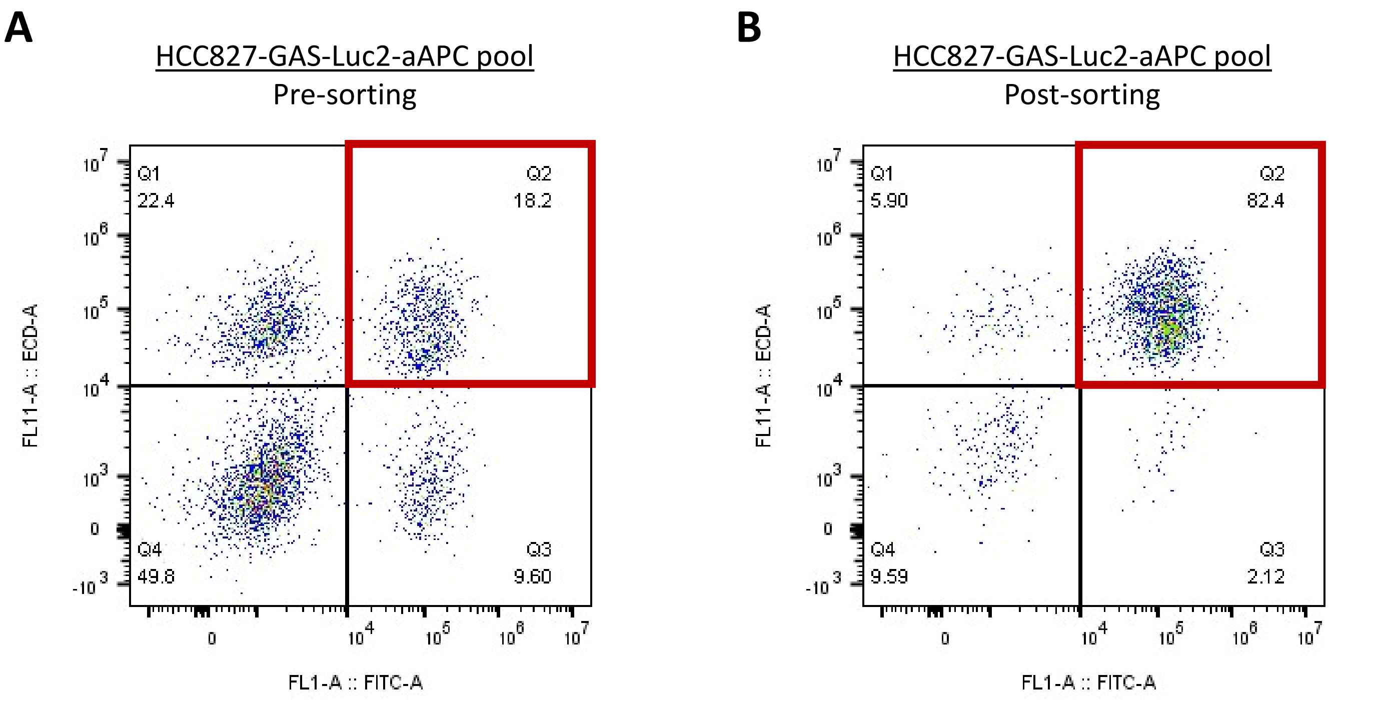

Supplemental Fig. S7. Flow cytometry analysis of HCC827-GAS-Luc2-aAPC multi-clone pool. The expression of the fluorescent markers on HCC827-GAS-Luc2-aAPC multi-clone pool before (A) and after (B) bulk sorting for GFP+/mCherry+ cells.
